# NANOGP8 is the key regulator of stemness, EMT, Wnt pathway, chemoresistance, and other malignant phenotypes in gastric cancer cells

**DOI:** 10.1371/journal.pone.0192436

**Published:** 2018-04-24

**Authors:** Xia Ma, Bei Wang, Xiaofang Wang, Yujiao Luo, Wufang Fan

**Affiliations:** 1 Molecular Biology Lab of Gastric Cancer, School of Life Sciences, Hebei University, Baoding, Hebei Province, China; 2 Key Laboratory of Medicinal Chemistry and Molecular Diagnosis of Ministry of Education, Hebei University, Baoding, Hebei Province, China; National Cancer Center, JAPAN

## Abstract

**Background:**

Accumulating evidence demonstrated that NANOG1, the key transcription factor for embryonic stem cells, is associated with human cancers. NANOGP8, one of the pseudogenes in NANOG gene family, contains an intact open reading frame and also said to be expressed in cancer tissues. Therefore, a systematic study is greatly needed to address the following questions: among NANOG1 and NANOGP8, which gene is the main contributor for NANOG expression in cancer cells and which one is the key regulator responsible for stemness, epithelial-mesenchymal transition (EMT), metastasis, chemoresistance and other malignant phenotypes. Here we try to explore these issues with gastric adenocarcinoma cell lines *in vitro* using variety of molecular and cellular techniques.

**Methods:**

Special primers were designed to distinguish PCR products from NANOG1 and NANOGP8. Sphere-forming cells were cultured with serum-free and selective medium. A stable cell line was established with infection of lentivirus containing NANOGP8. qPCR was performed to measure NANOGP8 expression and its association with stemness, EMT and CSC markers in adherent cells and sphere-forming cells. Western blot analysis was deployed to confirm results of the transcript analysis. Experiments of cell proliferation, migration, invasion, clonogenic assay, sphere cell growth assays, cell cycle analysis, β-catenin accumulation and translocation in nucleus, and drug resistance were conducted to measure the impact of NANOGP8 on malignant statuses of gastric cancer cells. Immunofluorescence staining was used to analyze cell subpopulations with different markers.

**Results:**

NANOGP8 is mainly responsible for NANOG expression in sphere-forming (stem cell-like) cells derived from gastric cancer cell lines regardless their differentiation status. Ectopic expression of NANOGP8 significantly up-regulates stemness transcription factors, EMT inducers, and cancer stem cell markers (CSC) including Lgr5. NANOGP8 also promotes expression of the signature genes vimentin and N-caderin for mesenchymal cells and down-regulates the signature gene E-caderin for epithelial cells whereby confer the cells with mesenchymal cell phenotype. In NANOGP8 over-expressed adherent and sphere-forming cells, Lgr5+ cells are significantly increased. Ectopic expression of NANOGP8 endows gastric cells with enhanced proliferation, migration, invasion, sphere-forming and clonogenic capacity, and chemoresistance. NANOGP8 expression also enhances β-catenin accumulation in nucleus and strengthens Wnt signal transduction.

**Conclusion:**

NANOGP8 is the main regulator of gastric cancer stem cells. It is closely associated with EMT, stemness, and CSC marker as well as Wnt signal pathway. NANOGP8 is correlated with cell proliferation, migration, invasion, clonogenic capacity, β-catenin accumulation in nucleus, and chemoresistance in gastric cancer. NANOGP8 is a promising molecular target for clinical intervention of gastric cancer.

## Introduction

Gastric cancer (GC) is the fourth most common cancer and the second leading cause of cancer death globally [1]. About 1 million new cases were diagnosed and more than 700,000 patients were died yearly, therefore, GC poses a big socioeconomic burden worldwide. In the past decade, despite the GC incidence rate is declining in western countries, but mortality rate is still high in Asia [2, 3]. Difficulty with early diagnosis and intrinsic resistance to chemotherapy may account for the bad outcomes. Until now, little is understood about its molecular etiopathogenesis, genetic risk of susceptibility as well as somatic drivers of GC progression.

Cancer stem cell is a recently proposed hypothesis. It proposes that only a small portion of the cancer cells, i.e., cancer stem cells (CSCs), is responsible for cancer initiation and progression [4]. CSC possesses both self-renewal and pluripotency capabilities. It is believed that CSC is originated from deregulated stem cells or dedifferentiated progenitor cells because normal stem cells and CSC shared the same stemness factors such as NANOG, OCT-4 and SOX2. These so-called core transcription factors not only play an indispensable role in embryonic stem cell (ESC) but also could reprogram the somatic cells back to an ESC-like state as showed by induced pluripotent stem Cell (iPC) [5, 6, 7]. The iPC fact suggests that the same ESC stemness factors with aberrant expression could be involved in cancer initiation and progression. In fact, up-regulated expression of Nanog, Oct-4 and Sox2 have been reported in many types of cancers [8]. In addition, increasing data demonstrated that CSCs are a group of cells possessing both features of stemness and epithelial-mesenchymal transition (EMT) [9]. EMT is known to endow cancer cells with metastasis potential. More and more evidences indicate that epithelial tissues originated CSCs generally express a mix features of epithelial and mesenchymal cells, suggesting mechanisms modulating stemness and EMT are closely coupled together [10, 11]. If it is the case, a stemness factor should play a key role for both CSC and EMT initiation and tightly associated with many tumor malignant phenotypes such as cancer cell proliferation, motility, evasion, metastasis, and drug resistance.

NANOG is a gene family containing 12 members, i.e., one prototype gene NANAOG1, one duplicated gene NANOG2, and 10 pseudogenes or retrogenes from NANOGP1 to NANOGP10 [12, 13]. It is believed that NANOG1(NM_024865.3) is only expressed in embryonic stem cells and play an indispensable role in maintaining stemness and pluripotency of ESCs; the retrogene NANOGP8(Gene ID:388112) is unique for human being, and is the only member of NANOG retrogene group that contains an intact open reading frame and is expressed. Until now, NANOGP8 is found to be only expressed in cancer tissues such as prostate, hepatocellular carcinoma, leukemia, glioblastoma multiforme, colorectal cancers, pancreatic cancer, and lung cancer [14, 15, 16, 17, 18, 19, 20], suggesting it may play a key role for cancers.

There are a few reports about expression of NANOG1 and NANOGP8 in gastric adenocarcinoma [21, 22, 23, 24, 25]. These findings are mainly focusing on detection NANOG expression by immunohistochemistry, NANOG protein association with lymph node status, infiltration scope and differentiation stages of the cancer. Few papers present a systemic study on NANOGP8 about its association with CSCs, EMT, Wnt pathway, cancer cell proliferation, cell wound healing, cancer metastasis and chemoresistance. Here we report our experimental data about role of NANOGP8 in gastric cancer regarding all aforementioned issues.

## Materials and methods

### Cell lines and sphere culture

Human gastric adenocarcinoma cell lines SGC7901 (moderate differentiation), MGC803 (low differentiation), MKN45 (low differentiation), and HGC27 (undifferentiation) was obtained from Institute of Basic Medical Sciences under Chinese Academy of Medical Sciences, and human gastric epithelial cell line GES-1 was obtained from Laboratory of Genetics at Beijing Cancer Hospital. All the cell lines were maintained in high glucose DMEM or RPMI-1640 (Solarbio, China) with 10% fetal bovine serum (FBS) (Transgen Biotech, China), 100IU/ml penicillin G and 100μg/ml streptomycin at 37°C in a humidified 5% CO_2_ incubator (Heal Force, Shanghai, China). For sphere formation, cells were collected and washed, then suspended in serum-free DMEM or RPMI-1640 culture medium containing 1% N-2 (17502–048, Gibco, USA) and 2% B-27 supplements (17504–044, Gibco, USA); 100U Penicillin /Streptomycin mixture (Shijiazhuang Pharmaceutical Group Co., Ltd, China), 20ng/ml human Fibroblast Growth Factor-basic (bFGF, FGF-2) (GF003, Millipore, Temecula, CA, USA) and 100 ng/ml Epidermal Growth Factor-basic (EGF) (GF144, Millipore, Temecula, CA, USA), and subsequently cultured in ultra low attachment 6-well plates (Corning Inc., Corning, NY, USA) at a density about 5,000 cells per well for 14 days per generation.

### Lentivirus preparation and infection

Lentiviral plasmid vectors expressing NANOGP8 (LV5-NANOGP8) and contrast LV5-NC were purchased from GenePharma (Suzhou, China). In detail, NANOGP8 gene is cloned into LV5 vector at NotI and NsiI restriction sites, and the LV5-NC is the same but without NANOGP8 insert. Note that Lentiviral NANOGP8 was corresponded to chemical synthesized *NANOGP8* gene sequence according to the NCBI Reference Sequence: NC_000015.10. The virus-containing supernatants were prepared according to the manufacturer’s instructions. For cell infection with the lentiviruses, SGC7901 cells were incubated with virus-containing supernatants at MOI = 20. Cells infected with the NANOGP8 viruses or the control viruses were selected in the presence of 2 ug/ml puromycin. To easily confirm >90% of cells were infected, the GFP -expressing viruses were used to infect the cells.

### qPCR and primers

Total-RNA was extracted from the parental cells and sphere-forming cells using RNAiso Plus (Takara Bio Inc., Japan) according to the manufacturer’s instructions. Reverse-transcription reaction to transcribe 2μg total-RNA into cDNA was performed with TransScript One-Step gDNA Removal and cDNA Synthesis Super Mix (Transgen Biotech, China). To determine fold changes in each gene, real-time qPCR was performed using SYBR Premix Ex TaqII PCR kit (Takara Bio Inc., Japan) in an Applied Biosystems 7500 Real-Time PCR System. The reaction mixture of 20 μl contained 10μl of SYBR Premix Ex TaqII PCR mix (Takara Bio Inc., Japan), 2 μl of primers (10mM) and 8 μl of template cDNA (0.4 μg). The *GAPDH* gene served as an internal control. The primer sequences are summarized in Table 1. After an initial incubation for 3 min at 95°C, the reactions were carried out for 39 cycles at 95°C for 20 sec and 60°C for 30 sec (fluorescence collection). Reactions with no template were included as negative control. By setting the threshold at the level at the middle steady portion of reaction cycles versus florescence curve, the Ct values of target genes were calculated using 7500 system SDS 1.4 software, and the 2(-ΔΔC(T)) method was used [26]. The qPCRs were performed three times in triplicate for each pair of primers against each sample.

**Table 1:** Primers used in this study.

Gene	Accession number	Primers	Tm	Product length
LGR5	NM-003667.3	5'-TCTGGTGAGCCTGAGAAAGC-3' 5'-ATGCTGGAGCTGGTAAAGGT-3'	57.45 55.40	138bp
CD44	NM_000610.3	5'-AGAGGCTGAGACAGGAGGTT-3' 5'-GCTTCCAGAGTTACGCCCTT-3'	57.45 57.45	256bp
CD133	NM_001145847	5'-ATTCACCAGCAACGAGTCCT-3' 5'-TTGCACGATGCCACTTTCTC-3'	55.40 55.40	269bp
CD54	NM-000201.2	5'-ACACTAGGCCACGCATCTG-3' 5'-TCATGGTGGGGCTATGTCTC-3'	57.90 56.80	137bp
TWIST	NM-000474.3	5'-GCCGGAGACCTAGATGTCATT-3' 5'-CCCACGCCCTGTTTCTTTGA-3'	57.8 57.45	151bp
PRRX1	NM_022716	5'-TGGAGCTTGAAGAGAATGGCT-3' 5'-TTCAGGCTTTGCTGTTTGCC-3'	55.85 55.40	174bp
Zeb	NM_001174094	5'-CCAACCCGTGCTAACTACCT-3' 5'-CCCTGCAATCAGAACTCAATGG-3'	57.45 57.80	198bp
E-cad	NM-004360.4	5'-TGTAACTTGCAATGGGCAGC-3' 5'-CAAGCTCTCCTGCCATCTCC-3'	55.40 59.50	178bp
Vimentin	NM-003380.4	5'-ACGTCTTGACCTTGAACGCA-3' 5'-TCTTGGCAGCCACACTTTCA-3'	55.40 55.40	200bp
N-cad	NM_001792.3	5'-GGGCCACGGTTCAAGAAACT-3' 5'-AGCCCAAATTGGTTTGCAGC-3'	57.45 55.40	199bp
NANOG1-S	NM_024865.3	5'-TTCATTATAAATCTAGAGACTCCAGGA-3' 5'-CTTTGGGACTGGTGGAAGAATC-3'	56.34 58.13	439bp
NANOG1/P8	NM_024865.3* NM-001355281*	5'-GCAGAGAAGAGTGTCG-3' 5'-AGCTGGGTGGAAGAGAACACAG-3'	59.60 49.30	82bp
ABCG2	NM_004827	5'-GGAATCTGACCACTCCTGGAT-3' 5'-GTGACTGGGAGAATGGCTGA-3'	56.40 57.20	304bp
MSI1	NM_002442	5'-GAACCATCCCGTCCTGTATCA-3' 5'-GAAACCATGAAGCCCCAACC-3'	56.60 57.10	280bp
GAPDH	NM-001289745.2	5'-AAGAAGGTGGTGAAGCAGGC-3' 5'-GTCAAAGGTGGAGGAGTGGG-3'	58.20 58.40	114bp

**Note:** The E-cad stands for E-caderin, N-cad stands for N-caderin, NANOG1-S stands for NANOG1-specific primers, and NANOG1/P8 stands for the common primers shared by both NANOG1 and NANOGP8.

* indicates that this set of primers matches to both NM_024865.3* and NM-001355281* sequences.

### Protein extraction and western blotting

Total proteins were extracted with a lysis buffer. The concentration of proteins in the supernatant was analyzed using the BCA method (Beyotime, China). Protein samples (60ug per lane) were loaded onto 12% SDS-polyacrylamide gels and electrophoresed, and then transferred to nitrocellulose membranes that were first blocked with 10% (wv^-1^) non-fat milk in TBST (50mM Tris-HCl pH 7.6, 150mM NaCl, 0.1% Tween-20) at room temperature for 1h and then incubated with a primary antibody diluted in TBST (anti-Lgr5, 1:1000; anti-NANOGP8, 1:800, OriGene, Rockville, MD; anti-GAPDH, 1:5000; anti-E-cad 1:1000, Proteintech, USA; anti-N-cad, 1:500, Invitrogen, USA; anti-Vimentin, 1:500, Calbiochem, USA; anti-CD44, 1:500, Abcam, UK) overnight at 4°C. After washing three times with TBST, membranes were incubated with a peroxidase-conjugated Affinipure Goat anti-mouse IgG or Goat anti-rabbit IgG (1:5000, proteintech, USA) for 1.5h at room temperature. Bands were visualized and quantified using the ECL chemiluminescence detection system (FluorChem E, Alpha, USA).

### Immunofluorescence staining

Mechanically dissociated tumor sphere-forming cells or adherent cells were fixed in 4% paraformaldehyde (Solarbio, China) for 30 min at room temperature. Cells were washed three times with PBS and incubated for 1 hour in 1% BSA blocking buffer which contained 10% goat serum, then incubated with anti-NANOGP8 rabbit monoclonal antibody (AP55015SU-N, OriGene, Rockville, MD) at 1:800 dilution and anti-LGR5 mouse monoclonal antibody (TA503316, OriGene, Rockville, MD) at 1:400 dilution, or anti- β-catenin rabbit monoclonal antibody (ab32472, Abcam, UK) overnight at 4°C. After three 10-min washes with PBS, the cells were incubated with the secondary antibody, FITC-conjugated goat anti-Rabbit and anti-Mouse IgG antibody (SA00003-1, Proteintech, USA) at 1:100 dilution in 1% BSA blocking buffer for 30 min at 37°C. After three 10-min washes with PBS, the cells were incubated with 0.1 μg/ml DAPI (Sigma, USA) at room temperature for 10 min. The cells were then washed for three 5-min with PBS, the localization and expression of NANOGP8 and Lgr5 were observed with an Olympus fluorescence microscope (Olympus, Japan). To detect nucleus-located of β-catenin, laser scanning confocal microscope was used (Olympus, Japan).

### Wound healing assay

A wound healing assay was performed to examine cell migration. Briefly, after the cells grew to 90% confluence in six-well plates, a single scratch wound was created by scraping with a 200 μl disposable pipette tip and then the debris was washed with PBS. Photographs were taken at indicated time-points to assess the ability of the cells to migrate into the wound area. The scratch wounds were photographed using a Nikon inverted microscope with an attached digital camera, and the widths of the wounds were quantified using Image software. Experiments were carried out in triplicate at least three times.

### Transwell Boyden chamber assay

Transwell Boyden chamber assay was performed in 24-well 8μm pore size transwell plates according to the manufacturer’s instructions (Corning, New York, NY, USA). The bottom of the transwell chamber was coated with BD Matrigel Basement Membrane Matrix (BD Biosciences, San Diego, CA, USA) in invasion assays. The upper chamber was full with 2×10^4^ cells in 200 μl of RPMI-1640 medium (Solarbio, China) containing 0.1% BSA. The lower chamber was full with 500 μl of RPMI-1640 containing 20ng/mL EGF (GF144, Millipore, Temecula, CA, USA) as a chemoattractant. After 24 h of incubation at 37°C, non-invading cells were removed from the surface of the membrane by scrubbing the upper side of the transwell and invading cells were fixed with methanol and stained with crystal violet. Six random fields on average were counted using a light microscope. Three independent invasion assays were performed in triplicate.

### Cell proliferation assay

MTT Counting Kit (Vazyme, Nanjing, China) was used to measure the cell proliferation. After the transfection, cells were seeded at the density of 5000 per well in 96-well microplates. Cell proliferation was examined after 1, 2, 3, 4, 5, 6, 7 days. 20μL of MTT solution was added to each well and incubated for 4 h at 37°C away from light. After the supernatant was removed, 150μL of DMSO solution was added to each well and shook for 10 minutes. The optical density was detected at a wavelength of 490 nm by microplate reader (SpectraMax M4, Molecular Devices, USA). Each sample had three replicates.

### Clonogenic assay

Adherent cell growth was assessed by cell counting at the indicated times. A total of 2× 10^4^ cells were seeded in 6-well plates, and colonies consisting of at least 50 cells were counted after 14 days, the cells were fixed by methanol about 15 minutes, stained by Gimsa dye for 10 minutes, then dried and photographed after washing. The number of colonies derived from NANOGP8-overexpressed cells is compared with the number of colonies derived from NANOGP8-mock cells. Experiments were carried out in triplicate at least three times.

### Sphere-forming capacity assay

Each cell line was plated at 5000 cells/well in ultra low attachment 6-well plates (Corning Inc., Corning, NY, USA) and cultured sphere formation medium, RPMI-1640 medium containing 1% N-2(17502–048, Gibco, USA), 2% B-27 supplements (17504–044, Gibco, USA), 100U Penicillin /Streptomycin mixture (Shijiazhuang Pharmaceutical Group Co., Ltd.), 20ng/ml human Fibroblast Growth Factor-basic (bFGF, FGF-2) (GF003, Millipore, Temecula, CA, USA) and 100 ng/ml Epidermal Growth Factor-basic (EGF) (GF144, Millipore, Temecula, CA, USA). After 14 days, the numbers of spheres for the NANOGP8 over expression cell lines were counted and compared with the number of spheres of NANOGNC cell lines. Each sample had three replicates.

### Drug resistance

Cell viability was assessed by an MTT assay after oxaliplatin (L-OHP, Nanjing Pharmaceutical, China) administration. NANOGP8 over-expression and NANOGNC was seeded in a 96-well plate at 1× 10^4^ cells/well; 24 hours later, L-OHP was added at concentrations of 15, 30, 60, or 100 uM/L. Cell viability was evaluated after 48 hours of L-OHP treatment.

### Flow cytometry

The cell cycle on SGC7901 with NANOGP8 transfected and NANOGNC cell lines was assessed by flow cytometry using Cycletest Plus DNA Kit (Becton, Dickinson and Company, US). The cells were washed twice in PBS and then labeled with PI stain solution by incubation away from light for 10 min on ice. The flow cytometry analysis was done with BD FACS Calibur^™^ (Becton, Dickinson and Company, US).

### Statistical analysis

Data are expressed as the mean ± standard deviation of three independent experiments. Statistical significance was determined with a paired t-test for two groups using SPSS 16.0 software (SPSS, Inc., Chicago, IL, USA). Two-sided tests were used to evaluate comparisons between two groups. P<0.05 was considered to indicate a statistically significant difference.

## Results

### NANOGP8 expression is up-regulated in sphere-forming cells

**Fig 1:**
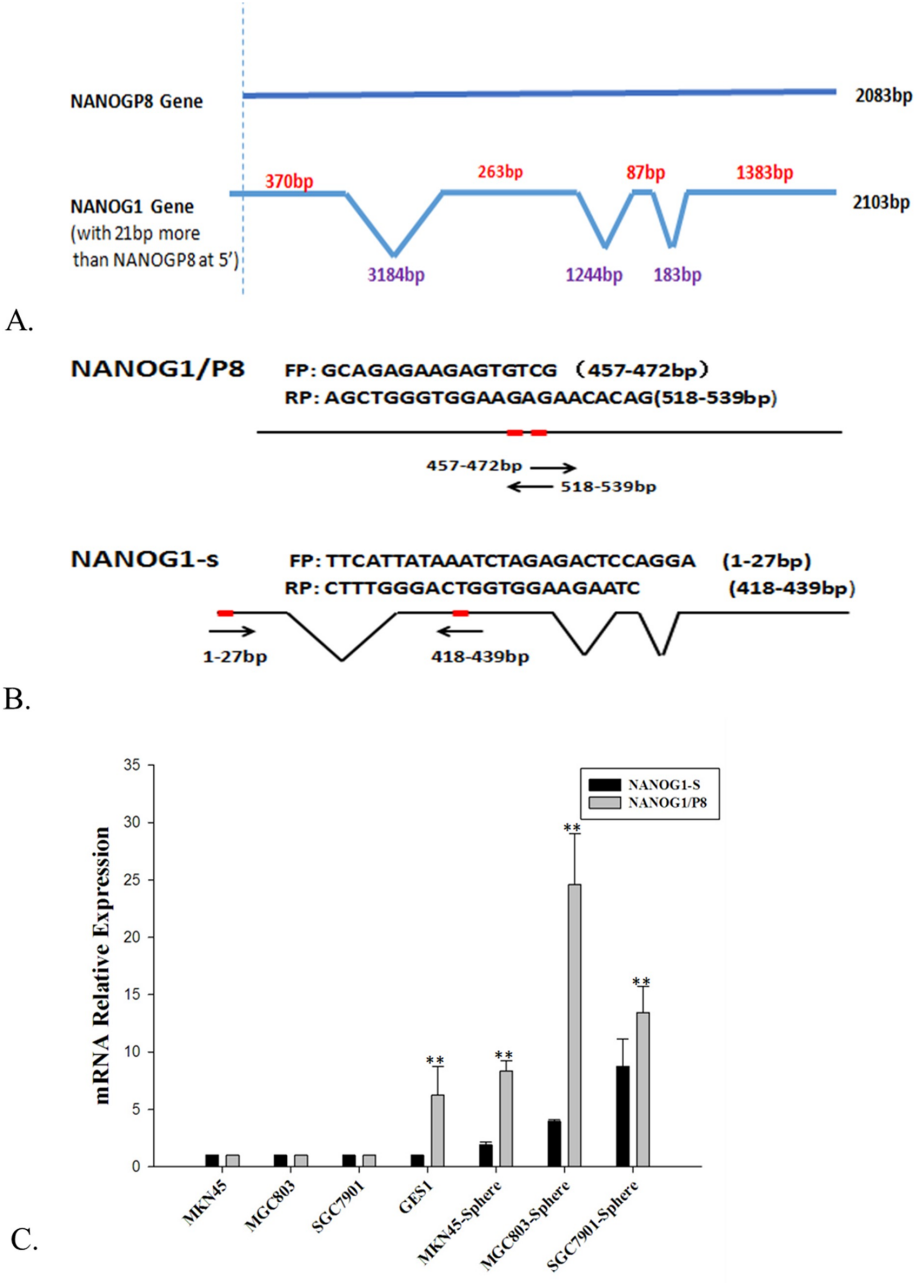
Detection of *NANOG-1* and *NANOGP8* expression by qPCR. **A.** Diagram of the gene structures of NANOG-1 and NANOGP8, note that the most 5’ of the first exon in NANOG1 gene contains a stretch of 21nt unique sequences; **B.** Diagram of the sequence locations of the primers designed to distinguish PCR products amplified from NANOG1 and NANOGP8. NANOG1/P8 stands for common primers shared by NANOG1 and NANOGP8 that can amplify DNA from both NANOG1 and NANOGP8 cDNA, and NANOG1-s stands for NANOG1-specific primers that only can amplify DNA from NANOG1 cDNA; **C.** Expression of NANOG1 and NANOG1/NANOGP8 in sphere-forming cells versus parental adherent cells derived from different cell lines. **p<0.01.

To explore *NANOGP8* expression in CSC cells, we enriched gastric CSCs with sphere –forming cell culture using MGC803, MKN45 and SGC7901 cell lines with various differentiation statuses. Then we performed qPCR to compare *NANOGP8* expression in sphere-forming cells and the parental adherent cells respectively. Since *NANOG1* mRNA possesses a unique stretch of 21 nucleotides at its most 5’, so we use this feature to design primers to distinguish PCR products amplified from NANOG1 and NANOGP8 (Fig 1A and 1B). The result shows that *NANOGP8* expression is significantly higher than *NANOG1* in all sphere-forming cells even for the GES-1 (human embryonic gastric epithelial cell) cell line (Fig 1C).

### NANOGP8 is associated with up-regulated EMT inducer genes and putative gastric cancer stem cell markers in gastric cancer cell line

**Fig 2:**
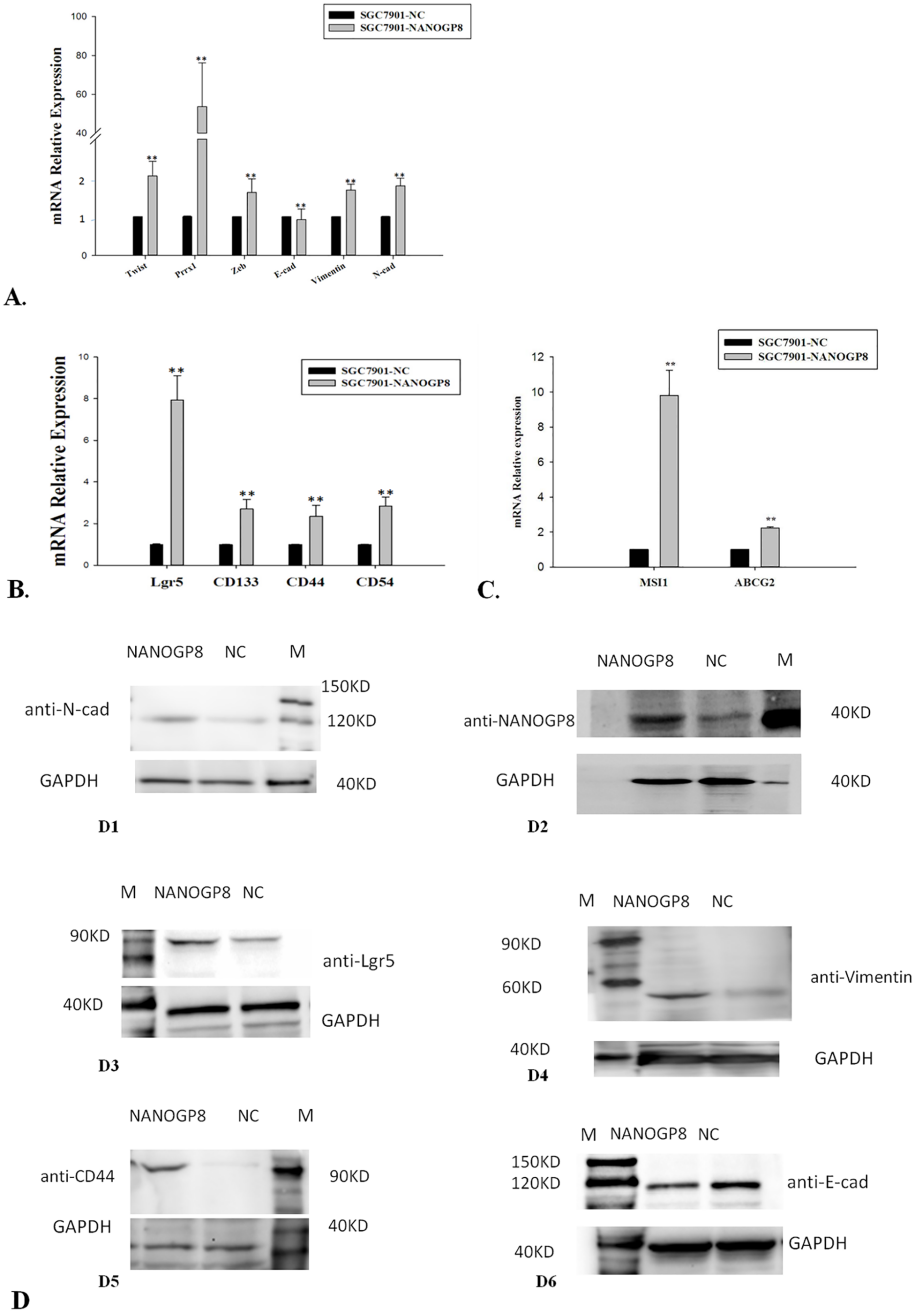
NANOGP8 over-expression enhances EMT and CSC Markers. **A.** Comparison of EMT genes in NANOGP8-transfected SGC7901 cells (SGC7901-NANOGP8) and mock-transfected SGC7901 cells (SGC7901-NC). **p<0.01. **B.** Expression of CSC marker genes in NANOGP8-transfected SGC7901 cells and mock-transfected SGC7901 cells. **p<0.01. **C.** Expression of ABCG2 and MSI1 genes in NANOGP8-transfected SGC7901 cells and mock-transfected SGC7901 cells. **p<0.01. **D.** Protein detection of EMT and CSC markers in NANOGP8-transfected SGC7901 cells (NANOGP8) and mock-transfected SGC7901 cells (NC). **D1.**Comparison of N-cad protein expression in NANOGP8-transfected SGC7901 cells (NANOGP8) and mock-transfected SGC7901 cells (NC); **D2.** Comparison of NANOGP8 protein expression in NANOGP8-transfected SGC7901 cells (NANOGP8) and mock-transfected SGC7901 cells (NC); **D3.** Comparison of Lgr5 protein expression in NANOGP8-transfected SGC7901 cells (NANOGP8) and mock-transfected SGC7901 cells (NC); **D4.** Comparison of vimentin protein expression in NANOGP8-transfected SGC7901 cells (NANOGP8) and mock-transfected SGC7901 cells (NC); **D5.** Comparison of CD44 protein expression in NANOGP8-transfected SGC7901 cells (NANOGP8) and mock-transfected SGC7901 cells (NC); **D6.** Comparison of E-cad protein expression in NANOGP8-transfected SGC7901 cells (NANOGP8) and mock-transfected SGC7901 cells (NC).

Since we observed that NANOGP8 expression is up-regulated in sphere-forming cells, then we ask if NANOGP8 has an impact on EMT property for gastric cancer cells. We transfected gastric cancer cell line MGC803 and SGC7901 with NANOGP8-carried lentivirus, but MGC803 is refractory to transfection, so we have to use transfected SGC7901 as experimental materials in later studies. At MOI = 20, the efficiency of lentivirus transfection of SGC7901 is about 90%. We performed qPCR to detect EMT core genes expression. The result shows that, in NANOGP8-transfected SGC7901 cells, the expression of PRRX1, TWIST, ZEB, Vimentin and N-CAD are significantly up-regulated (>2 folds), and the expression of E-CAD, the major signature gene for epithelial cells, is significantly down-regulated (Fig 2A). It is believed that EMT is coupled with cancer stem cell, therefore, we asked if NANOGP8 regulates putative gastric cancer stem cell markers. We chose a set of genes that were reported to encode gastric cancer stem cell markers such as LGR5, CD133, CD44, CD54, ABCG2 and MSI1, and then performed qPCR to detect their expression in both NANOGP8-transfected (SGC7901-NANOGP8) and mock cells (SGC7901-NC). The result shows that expression of LGR5, CD133, CD44, CD54, ABCG2 and MSI1 was significantly up-regulated in NANOGP8-transfected cells (Fig 2B and 2C), and Lgr5 and MSI1 are the highest ones (>8 and >10 folds respectively). The protein expression was analyzed by Western blot (Fig 2D) that was consistent with qPCR, suggesting NANOGP8 may be a major regulator for EMT and CSC in gastric cancer cells.

### Cellular localization of NANOGP8 and Lgr5 in gastric cancer cells and sphere-forming cells

**Fig 3:**
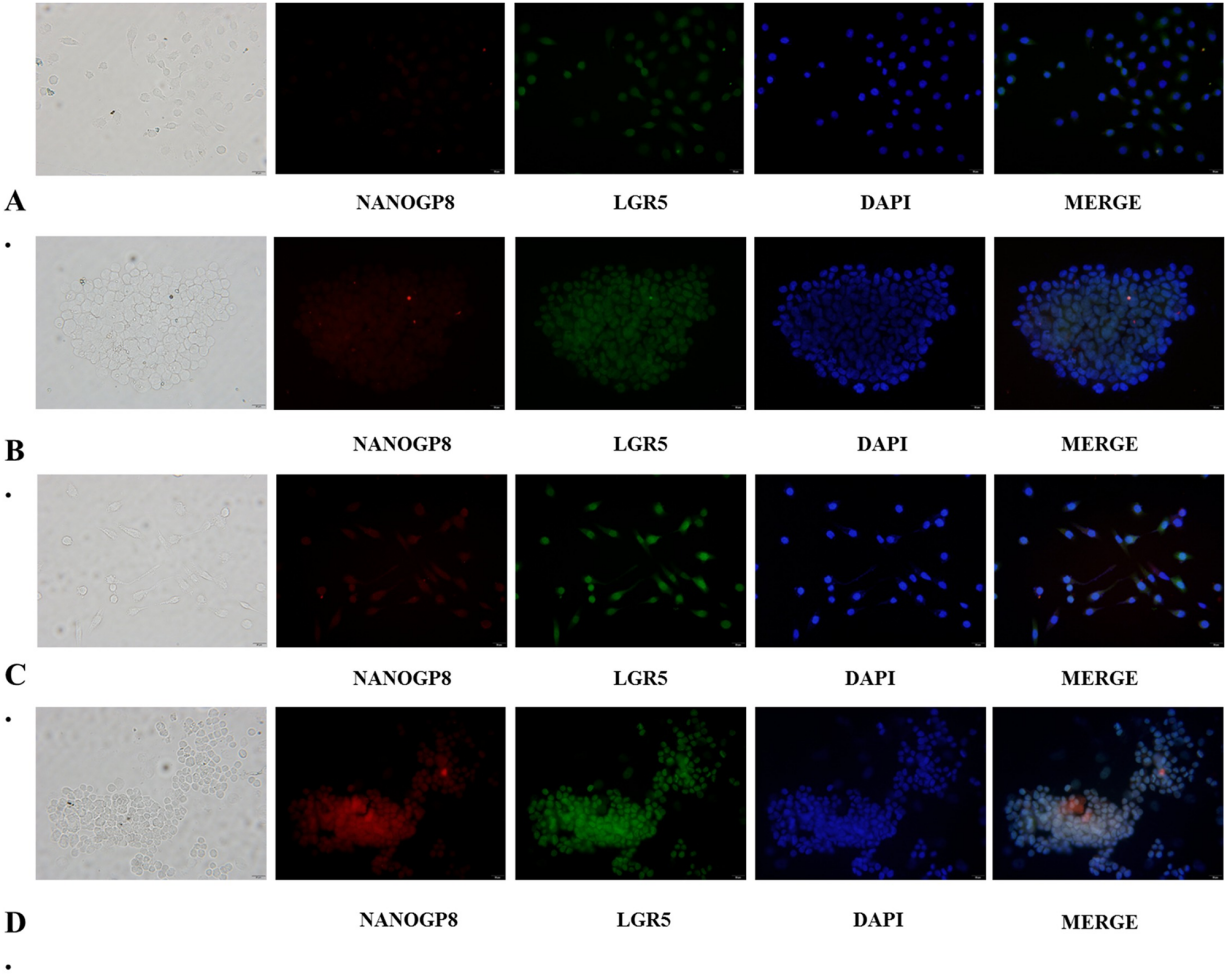
Immunofluorescence staining of LGR5 and NANOGP8 in SGC7901 cells and spheres. **A.** SGC7901 adherent cells; **B.** SGC7901 sphere-forming cells; **C.** NANOGP8-transfected SGC7901 adherent cells; **D.** NANOGP8-transfected SGC7901 sphere-forming cells. Note the localization and expression of NANOGP8 and Lgr5 were visualized. All the images are 200× magnified.

To visualize the localization of NANOGP8 and Lgr5 in gastric cancer sphere-forming cells and adherent cells, we performed immunofluorescence double staining with NANOGP8 and Lgr5 specific monoclonal antibodies. We found that in both SGC7901 sphere cells and adherent cells, NANOGP8 was localized in the nucleus or perinuclear region, while Lgr5 was localized in cell membrane or cytoplasm region. NANOGP8 enhanced fluorescence was found in SGC7901 sphere-forming cells compared to adherent cells (Fig 3A and 3B), this is consistent with our qPCR result. Furthermore, we observed that with increased NANOGP8 expression in NANOGP8-transfected cells and sphere cells, the LGR5 fluorescence was significantly boosted (Fig 3C and 3D). This result suggests that LGR5 was regulated by NANOGP8.

### NANOGP8 boosts accumulation and translocation of β-catenin in nucleus

**Fig 4:**
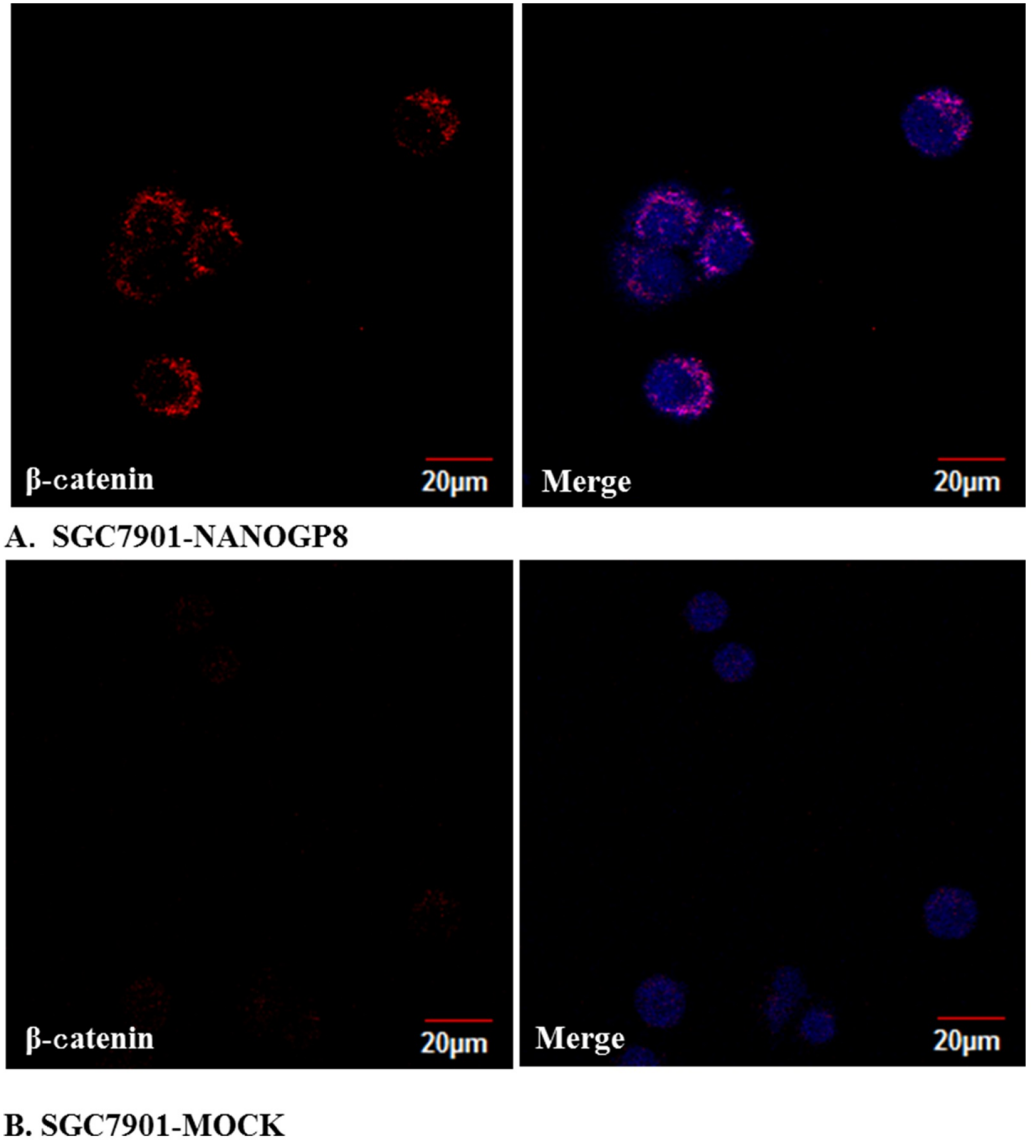
The fluorescence images of nucleus-located β-catenin in NANOGP8-transfected and mock cells. **A.** Accumulated β-catenin in nucleus of the NANOGP8-transfected cells (SGC7901-NANOGP8); **B.** Very weak or undetectable β-catenin in nucleus of the mock-cells (SGC7901-MOCK) without NANOGP8 transfection.

Because NANOGP8 promotes LGR5 expression the most significantly, and LGR5 is reported to be gastric cancer stem cell marker and one of the Wnt receptors, therefore, we want to explore if NANOGP8 can enhance Wnt signal transduction. We performed immunofluorescence staining with laser scanning confocal microscope to compare nucleus-located β-catenin in NANOGP8-transfected and mock cells. The result showed that β-catenin greatly translocated and accumulated in nucleus of NANOGP8-transfected cells while it is weak or even undetectable in nucleus of the mock cells (Fig 4A and 4B), suggesting NANOGP8 enhances Wnt signal transduction by promoting β-catenin accumulation in nucleus via up-regulated LGR5 expression, which in turn exerts impact on cell behaviors.

### Impact of NANOGP8 on malignant potential of gastric cancer cells

#### NANOGP8 over-expression promote cancer cell migration

**Fig 5:**
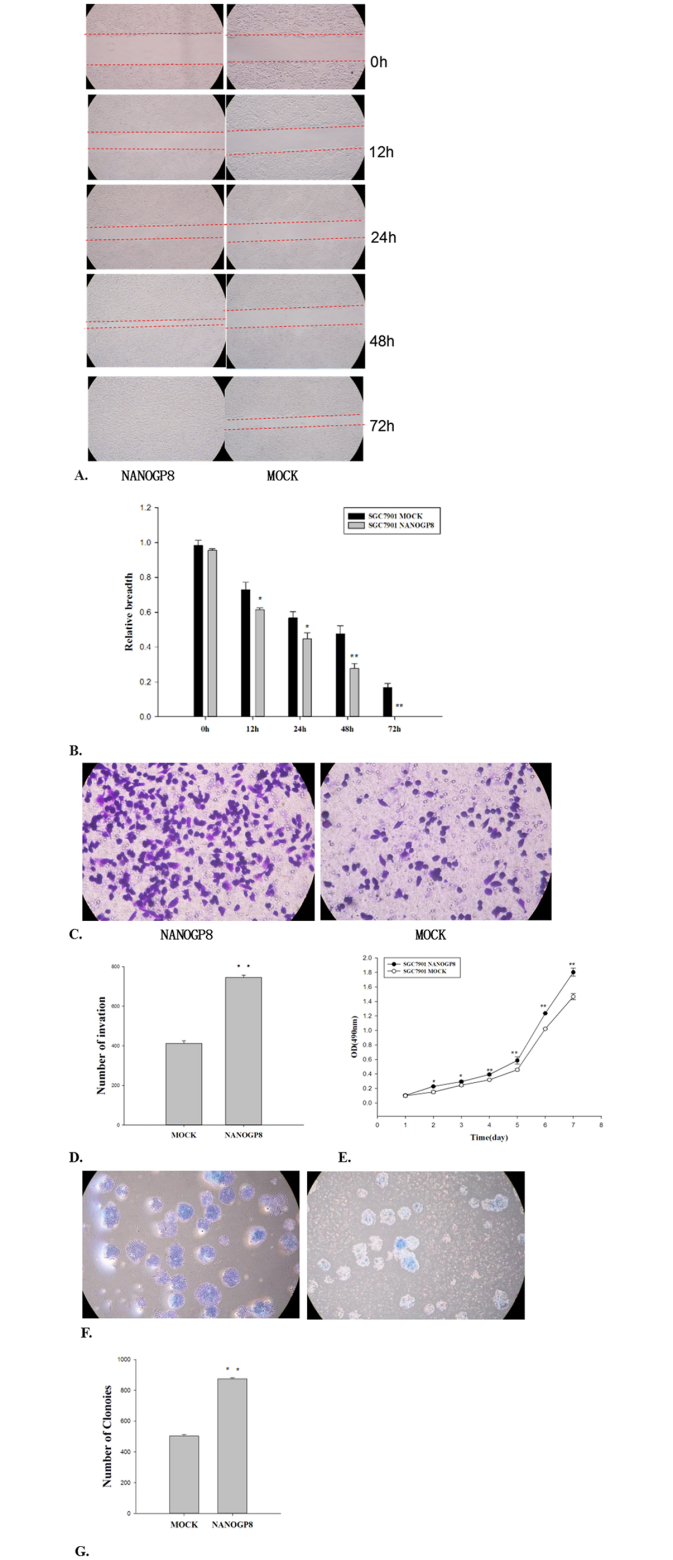
Impact of NANOGP8 on malignant phenotypes of gastric cancer cells. NANOGP8 stands for the NANOGP8 transfected SGC7901 cells, and mock stands for the mock transfected SGC7901 cells. All the experiments were repeated at least 3 times, and the results were statistically analyzed by a paired t-test for two groups using SPSS 16.0 software (SPSS, Inc., Chicago, IL, USA). P<0.05 was considered a statistically significant difference. **A.** Result of wound healing assay (cell migration). The cell wounds were visualized at 0 h, 12 h, 24 h, 48h, and 72 h; **B**. Histogram of wound healing assay at different time points. *p<0.05; **p<0.01. **C**. Result of transwell Boyden chamber assay. Note that the cell number passing the chamber membrane from NANOGP8-transfected cells is much more than that of mock-transfected cells; **D.** Histogram of the transwell Boyden chamber assay; **p<0.01. **E.** Result of cell proliferation assay *p<0.05; **p<0.01. **F.** Result of clonogenic capacity assay. The images show the Giemsa staining of clones at 14d; **G.** Histogram of clonogenic capacity assay, **p<0.01.

To explore if NANOGP8 expression is associated with enhanced cancer cell migration, we performed wound-healing assays in SGC7901 cell line after NANOGP8 transfection (Fig 5A). Comparison of cell migration between SGC7901-NANOGP8 and SGC7901 MOCK cells (Fig 5B). The migration distances from 0h to 72h for SGC7901-NANOGP8 are 0.955mm±0.0086; 0.6147mm±0.01012; 0.4467mm±0.03512; 0.2767mm±0.02887 and 0mm respectively; the migration distances from 0h to 72h for SGC7901-MOCK cells are 0.9833mm±0.02887; 0.7283mm±0.04311; 0.5667mm±0.03512; 0.4767mm±0.04509 and 0.1667mm±0.02517 respectively. The migration differences between 12h and 24h for these two groups are significant (P<0.05) and between 48h and 72h are extremely significant (P<0.01). The results (Fig 5B) showed that the cell migration speed was significantly enhanced in NANOGP8-transfected cells compared to mock cells (*P*<0.01).

#### NANOGP8 over-expression promote invasion/clonogenic/proliferation potential

To understand more about NANOGP8 association with tumor cell properties such as cell invasion, proliferation and self-renewal capacities, we performed transwell Boyden chamber assay, MTT experiment and clonogenic assay. Comparison of cell invasion potential between SGC7901-NANOGP8-transfected and mock cells by Transwell Boyden chamber assay (Fig 5C and 5D). The transmembrane cells from NANOGP8-transfected cells are significantly more than mock control cells (746±12 vs. 412±13) with an 81.07% of increase (P<0.01), which indicate that NANOGP8 over-expression promote invasion potential for gastric cancer cell line SGC7901. Comparison of cell proliferation between SGC7901-NANOGP8 and mock cells (Fig 5E). The proliferation results of day-1 to day-7 for SGC7901-NANOGP8 cells are 0.1049±0.0028; 0.2286±0.0037; 0.2943±0.01327; 0.3931±0.0108; 0.5864±0.04987; 1.2374±0.00132 and 1.8027±0.05565 respectively. The results of day-1 to day-7 for SGC7901-MOCK cells are 0.1009±0.01934; 0.1523±0.01507; 0.2460±0.01426; 0.3195±0.006; 0.4584±0.01252; 1.0227±0.00824 and 1.4667±0.0427 respectively. The proliferation differences between these two groups for day-2 and da-3 are significant (P<0.05), the differences between day-4 to day-7 are extremely significant (P<0.01). Comparison of SGC7901-NANOGP8 and mock cells by clonogenic assay (Fig 5F and 5G). The colony number formed by SGC7901-NANOGP8 are 875±7, the colony number formed by SGC7901 mock control are 503±8. The NANOGP8 shows an 73.96% of increase (P<0.01). The difference between these two groups is extremely significant (P<0.01). The results showed that the invasion (Fig 5C and 5D), proliferation (Fig 5E) and clonogenic capacity (Fig 5F and 5G) was significantly enhanced in NANOGP8-transfected cells compared to mock cells (*P*<0.01).

#### NANOGP8 enhances sphere formation

**Fig 6:**
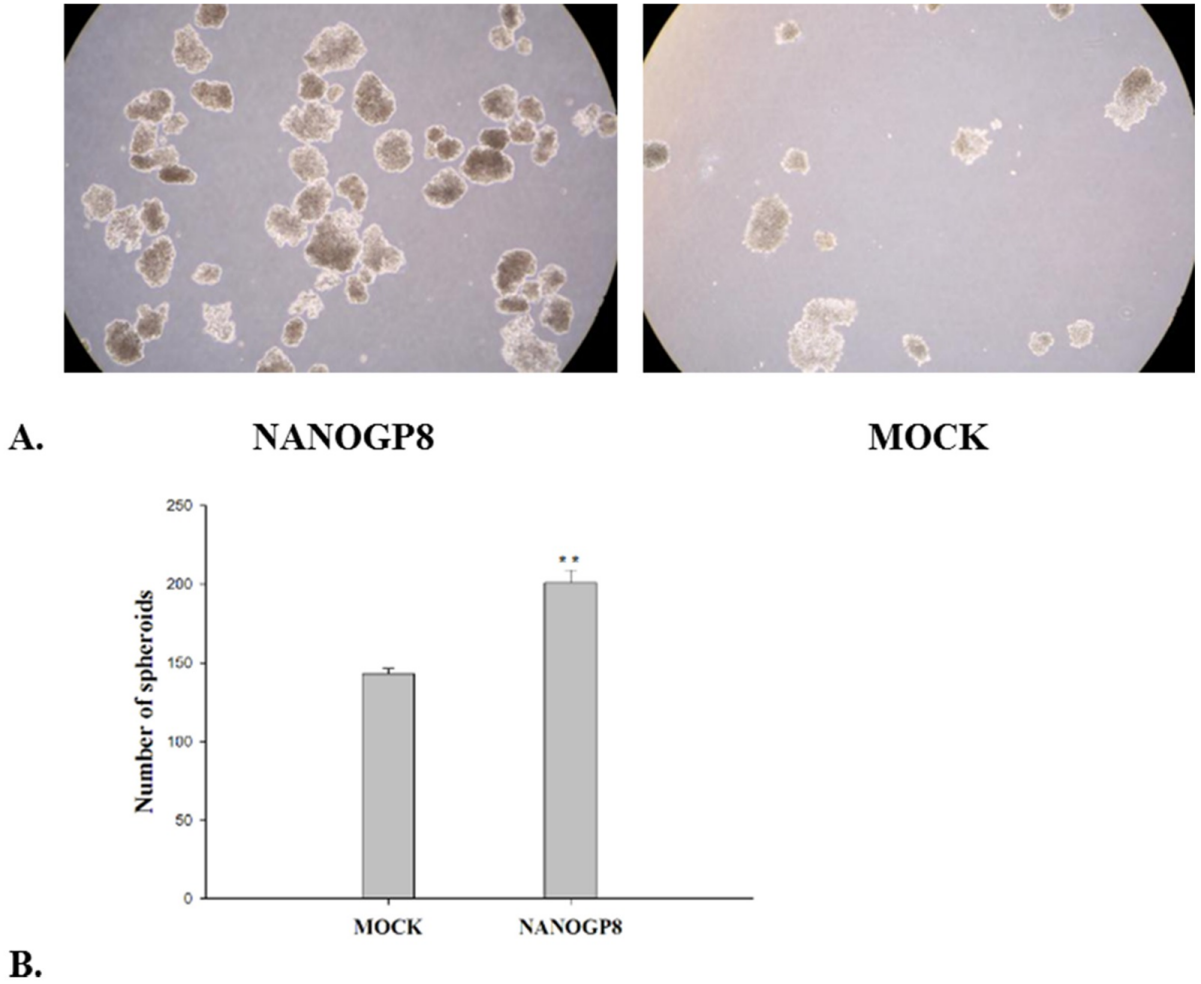
Result of sphere-forming cell growth assays in NANOGP8-transfected and mock cells. **A.** Images of the sphere-forming cell growth in the described condition for NANOGP8-transfected (NANOGP8) and mock cells (Mock). **B.** Histogram of sphere cells growth assay of NANOGP8-transfected and the mock cells. **p<0.01. The experiments described above were repeated at least 3 times, and the results were statistically analyzed by a paired t-test for two groups using SPSS 16.0 software (SPSS, Inc., Chicago, IL, USA). P<0.05 was considered a statistically significant difference. For details, please see the corresponding section in Materials and Methods.

To know if NANOGP8 can promote sphere formation, we did the sphere forming assay in NANOGP8 transfected cells and mock cells respectively. Comparison of sphere forming capacity by SGC7901-NANOGP8 and SGC-mock cells (Fig 6). The sphere cells were counted after the above described cells was incubated in a given medium for 14 days. The sphere cells from SGC7901-NANOGP8 are 201±8, and the sphere cells from SGC7901-mock cells are 143±3. The difference is extremely significant (P<0.01), indicating NANOGP8 over-expression can promote sphere-forming capacity for gastric cancer cell line. The results (Fig 6) showed that the sphere formation capacity is increased approximately 50% in NANOGP8-transfected cells compared to mock cells (*P*<0.01).

### NANOGP8 over-expression increases anti-oxaliplatin (L-OHP) resistance

**Fig 7:**
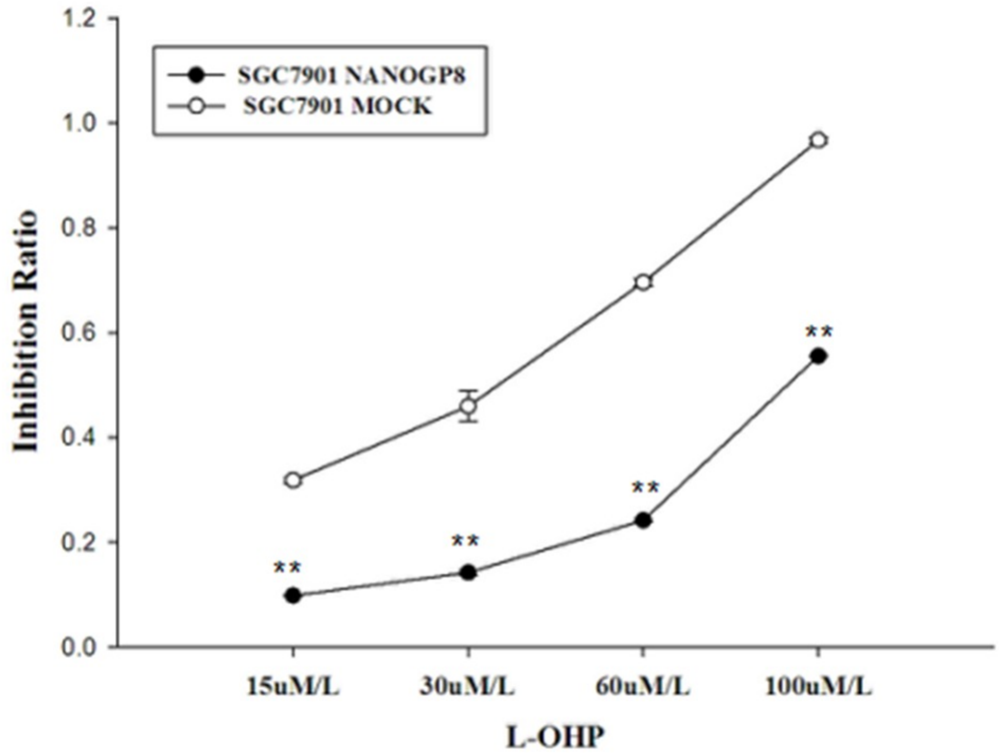
Result of chemoresistance assay. SGC7901 NANOGP8 stands for NANOGP8-transfected cells and mock stands for mock-transfected cells. **p<0.01. The experiments described above were repeated at least 3 times, and the results were statistically analyzed by a paired t-test for two groups using SPSS 16.0 software (SPSS, Inc., Chicago, IL, USA). P<0.05 was considered a statistically significant difference. For details, please see the corresponding section in Materials and Methods.

To explore if NANOGP8 influence drug sensitivity in gastric cancer cells, we examined drug sensitivity in NANOGP8 transfected and mock cells at different L-OHP concentration (15uM/L, 30uM/L, 60uM/L, 100uM/L) using an MTT assay. Comparison of chemoresistance of SGC7901-NANOGP8 and mock cells (Fig 7). Applying oxaliplatin (L-OHP) onto cells at different concentration (15uM, 30uM, 60uM and 100uM/ml respectively), and then the cells viability was assessed by an MTT assay after 48 hours incubation. The OD490nm values detected by a fluorescence ectrophotometer (Molecular Devices M4) were 0.0984±0.0015; 0.1426±0.0005; 0.2418±0.0029; and 0.5555±0.0010 respectively for SGC7901-NANOGP8 cells treated with different concentration of L-OHP, and the corresponding values for SGC7901-mock cells were 0.3185±0.0061; 0.4597±0.0297; 0.6960±0.0072; and 0.9670±0.0059 respectively. Statistical analysis shows extremely significant difference (P<0.01) of chemoresistance between these two cell groups at each drug concentration. The result showed (Fig 7) that NANOGP8 transfected cells significantly enhance drug resistance to L-OHP compared to mock cells.

### NANOGP8 regulates cell cycles in gastric cancer cells

**Fig 8:**
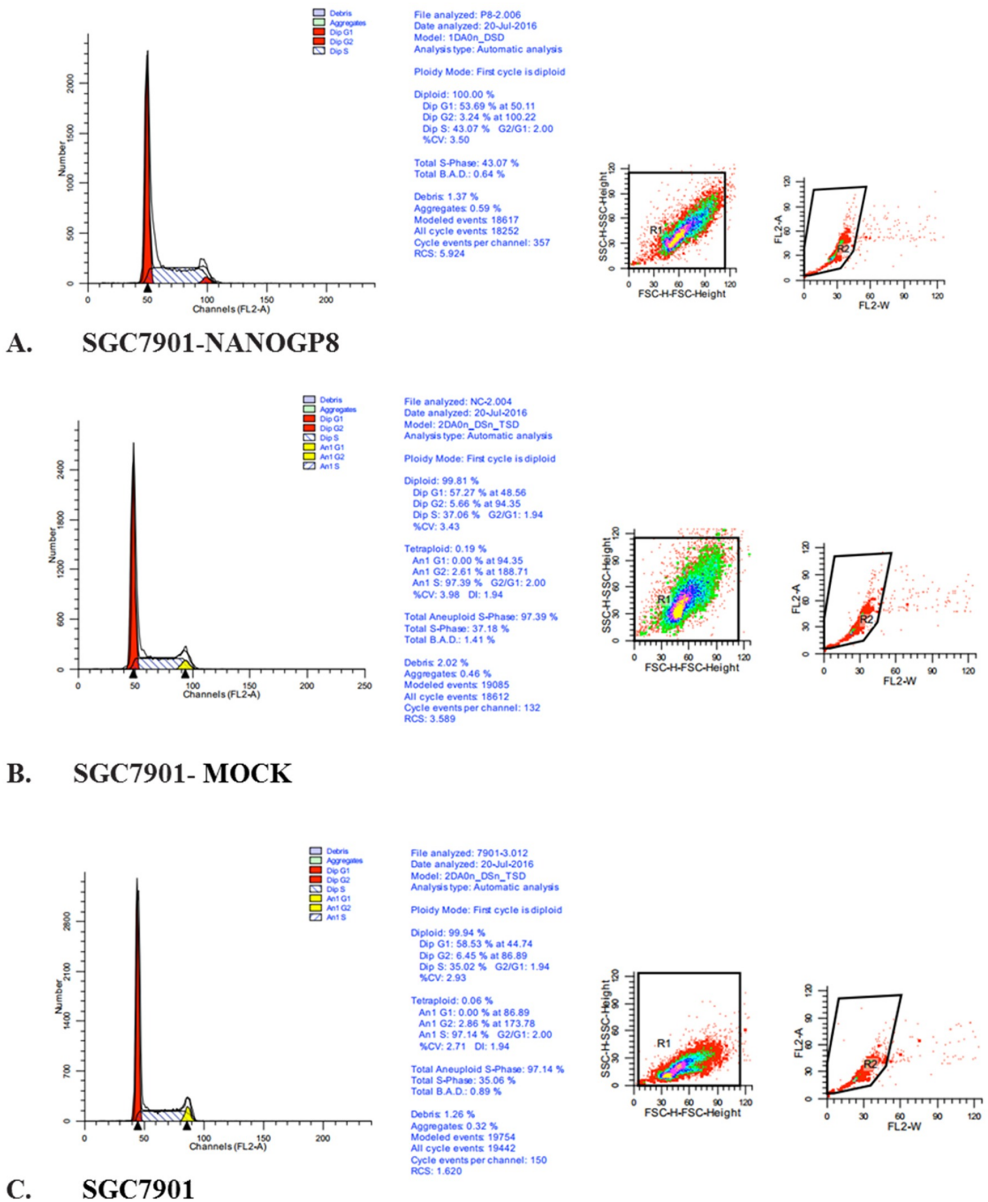
Flow cytometry analysis of cell cycle in NANOGP8-transfected, mock-transfected, and SGC7901 cells. **A.** Cell cycle in NANOGP8-transfected SGC7901 cells; **B.** Cell cycle in mock-transfected cells; **C.** Cell cycle in SGC7901 cells.

To understand if NANOGP8 exerts impact on cell cycle regulation, we performed flow cytometry and compared NANOGP8-transfected cells with mock and SGC7901 cells (Fig 8A–8C), we found that NANOGP8 have accelerated S-phase entry and reduced G0/G1 phase in NANOGP8-transfected cells (*P*<0.01) compared to the control cells (Table 2). This is consistent with our observation in cell proliferation assay.

**Table 2:** The impact of NANOGP8 on cell cycle in NANOGP8-transfected, mock and SGC7901 cells. **(%, mean ±** SD).

	G0-G1	G2-M	S
NANOGP8	54.45±0.89*^#^	4.23±1.02	41.31±1.90*^#^
NANOGNC	57.80±0.77	5.21±0.44	36.99±0.38
SGC7901	58.63±0.35	6.31±0.29	35.06±0.07

*P<0.01 vs NANOGNC,

^#^P<0.01 vs SGC7901.

## Discussion

Our results demonstrated that NANOG is highly expressed in gastric CSC, and NANOGP8 contributes for most of the NANOG expression. NANOGP8 expression is closely associated with up-regulated EMT transcription factors and down-regulated E-caderin. NANOGP8 expression also promotes gastric CSC markers such as Lgr5, CD44, CD133, CD54, MSI1 and ABCG2 especially for Lgr5 and MSI1; and enhances Wnt signal pathway by increasing β-catenin accumulation in nucleus. NANOGP8 expression significantly boosts cancer malignancy, such as cancer cell proliferation, migration (wound healing assay), invasion (transwell Boyden chamber assay), as well as strongly enhanced chemoresistance. NANOGP8 expression accelerates cell entry of S-phase and reduces cell numbers in G0/G1 phase. The results clearly show that NANOGP8 plays a key role in gastric cancer initiation, progression and malignancy, strongly suggests NANOGP8 is an excellent therapeutic target.

It is believed that NANOG1 control stemness in ESCs, NANOGP8 is only expressed in cancer tissues and plays a key role for CSCs. Our results are consistent with this proposal. Our data proved that NANOGP8 is the main contributor for NANOG expression in sphere-forming cells other tan NANOG1. We also observed that NANOGP8 is up-regulated in sphere-forming cells derived from GES1 cell line that was originated from mucous epithelium of embryo stomach. Considering GES1 is already a transformed cell line with pre-cancerous property [27], it is not surprised that NANOGP8 is up-regulated in it.

In NANOGP8 over-expressed cell line, we observed expression of several putative CSC markers such as LGR5, CD44, CD133 and CD54 were significantly erected, especially for Lgr5. Interestingly we found that the newly identified functional CSC markers, MSI1 and ABCG2, are also significantly up-regulated in NANOGP8 transfected cells, especially for MSI1. It has been established that MSI1 is a cancer stem cell marker and its expression is closely associated with stem cell proliferative and differential status [28]. Some studies demonstrated that MSI1 enhances epithelial cell growth by promoting canonical Wnt signaling pathway [29], which is consistent with our findings that NANOGP8 raises β-catenin accumulation in cancer cell nucleus (see Fig 4). It is believed that ABCG2 confer the side population phenotype and is recognized as a universal CSC marker that controls stem cell phenotypes such as multidrug resistance [30, 31]. These data are consistent with our findings that NANOGP8 confers gastric cancer cell with chemoresistance (see Fig 7). Combined with our results, it may suggests that NANOGP8 play its partial role via MSI1 and ABCG2 as components in its regulatory circuit. Our previous study showed that the number of LGR5+/CD54+ cells are greatly increased in sphere-forming cells compared to adherent cells [32]. Other groups reported that CD44 and CD133 are CSC markers of GC too. In any events, these facts showed that NANOGP8 regulates expression of CSC markers and promotes CSC phenotypes, indicating NANOGP8 is closely associated with CSC emergence and proliferation. Using immunofluorescent staining to detect intensity of NANOGP8+ and Lgr5+ cells in NANOGP8 over-expressed cells, the result further supports our observation (Fig 3). With ectopic expression of NANOGP8, Lgr5 protein accumulates immensely in the cells compared to mock transfected cells, in which Lgr5 specific immunofluorescent staining is weak or even undetectable (see result in Fig 3). Another phenomenon we observed is that, in NANOGP8 over-expressed cancer cells, expression of EMT signature genes such as TWIST1, PRRX1, ZEB, Vimentin, and N-caderin, was significantly up-regulates and expression of the epithelial marker E-caderin was reversely down-regulated. These results clearly demonstrated that NANOGP8 is a driver for EMT process, which induces conversion of epithelial cells into mesenchymal cells, thereby confers cancer cells with invasive and metastasis phenotypes. Yao et al reported NANOG can bind to Slug promoter, and form a NANOG/Slug/IGF/STAT3/ signaling axis and simultaneously controls EMT and stemness [33]. Our finding is similar to them. We believe that NANOGP8/TWIST/ZEB/PRRX1 may be the upstream regulators of EMT, and vimentin/N-caderin/E-caderin may be the structural components to endow cancer cells the EMT properties. Migita et al found that EMT can regulate stemness core factors in bladder cancer, and concluded that EMT and stemness is coupled together to form an EMT-cancer stemness axis [34]. Their observation supports our findings too. All the data strongly suggest that stemness and EMT may closely communicate each other and determine the cancer malignancy and disease outcome.

Cancer cells express many malignant phenotypes. We found that all the malignant phenotypes are associated with NANOGP8. Ectopic expression of NANOGP8 enhances CSC markers (Fig 2C), it also enhances cancer cell proliferation (Fig 5E), cell migration (Fig 5A & 5B), clonogenic capacity (Fig 5F), sphere-forming cells growth (Fig 6A). Clearly, NANOGP8 regulate CSCs, and CSCs endow the cancer cells with these malignant phenotypes. Furthermore, ectopic expression of NANOGP8 boosts expression of the EMT inducer genes, which in turn enhances cell invasive property such as transwell-Boyden chamber assay (Fig 5C & 5D), and chemoresistance (Fig 7). NANOGP8 up-regulate N-caderin, vimentin and down-regulate E-caderin, which makes the cells ready to express EMT phenotypes. It clearly indicates that NANOGP8 induces EMT, and EMT gene expression confers the cancer cells with EMT-associated malignant characteristics. It is well-accepted EMT is a crucial biological event that is involved in cell transformation, oncogenesis, metastasis and chemoresistance in many cancers [35]. EMT is characterized by loss of epithelial features and gain of mesenchymal features of the epithelial cells. Decreased expression of E-Cadherin and increased expression of N-Cadherin, Vimentin, PRRK1 and Twist are the major hallmarks of EMT process. Our results are completely consistent with these observations and strongly support this theory.

It has been reported that EMT induction was promoted by Wnt signaling pathway. We observed that ectopic expression of NANOGP8 extremely up-regulates Lgr5 in cancer cells (>8 folds), and in turn, it significantly enhances translocation/accumulation of β-catenin in cancer cell nucleus. We believe it may underlie one of the mechanisms how NANOGP8 regulates stemness and EMT. As we observed, β-catenin is heavily accumulated in nucleus of NANOGP8 over-expressed cancer cells, while it is undetectable in nucleus of the mock cells. This is perfectly consistent with the hypothesis raised by other authors who propose that Wnt is an important pathway to activate EMT [36, 37, 38]. There are evidences that ablation of the β-catenin gene can results in great loss of CSCs and complete tumor regression, furthermore, some data demonstrated that β-catenin is only increased in malignant cancer cells but it is not essential in normal epithelium [39]. So it is believed that β-catenin is essential to maintain CSC and it may be targeted to eliminate CSCs and finally eradicate all cancer cells. Yong et al reported that Helicobacter pylori can promote CSC by up-regulating Nanog and Oct4 via Wnt/β-catenin signaling pathway [40]. They showed that β-catenin can bind to NANOG promoter and activate NANOG and OCT-4 thereby to induce stemness. Our result supports NANOG and β-catenin interaction but in an opposite order. We showed NANOGP8 activates β-catenin but not reversal because forced expression Lgr5 in cancer cells had no impact on NANOG1/NANOGP8 expression. In contrast, the forced expression of NANOGP8 significantly up-regulates Lgr5 expression. This conflict may be due to differences between NANOG1 and NANOGP8 that don’t share the same promoters. We believe NANOGP8 enhances β-catenin entry of nucleus via promoting Lgr5. It is well- known that Lgr5 is one of the components in Wnt receptor complex and LGR5 promotes R-spondin-mediated Wnt/β-catenin and Wnt/PCP signaling [41]. As a CSC marker, Lgr5 supposes to be activated first by stemness controlling gene such as NANOGP8. We believe that expression of NANOGP8, up-regulation of Lgr5, and accumulation of β-catenin is a cascade event. This phenomenon surely suggests that NANOGP8, Lgr5 and β-catenin comprise a signal transduction axis, which is responsible for all the phenotype changes observed in the NANOGP8 over-expressed cancer cells.

Intrinsic resistance to chemotherapy is the most notorious feature of gastric cancer, it partly accounted for the high mortality and low survival rate [42]. We demonstrated that ectopic expression of NANOGP8 not only up-regulates EMT markers and enhances β-catenin accumulation in nucleus, but also confers cells with significantly higher resistance to chemotherapy oxaliplatin. It is said a pathway called EMT/β-catenin axis is responsible for cancer chemoresistance [43], our findings are perfectly consistent with this concept. It is believed that CSC stemness is associated with EMT, and EMT co-exists with chemoresistance, the anti-chemotherapy is merely an intrinsic feature of EMT. EMT association with chemoresistance have been observed in many other cancers such as pancreatic cancer, ovarian cancer, breast cancer, nasopharyngeal cancer and lung cancer [44, 45, 46, 47]. Our data first showed that NANOGP8 enhances EMT markers and Wnt signals, meanwhile endow the cancer cells with chemoresistance. Many factors may contribute to EMT/chemoresistance, even proton pump and glycolysis [42, 48], but we believe NANOGP8 is the essential driver for GC chenoresistance.

In summary, our data showed that NANOGP8 is the main contributor of NANOG expression in gastric CSC. Ectopic expression of NANOGP8 up-regulates CSC markers and EMT signature genes in gastric cancer cells, which, in turn, greatly enhance cell proliferation, migration, invasion, sphere cell growth, Wnt signaling and chemoresistance. Our systematic study clearly demonstrated that NANOGP8 is the essential regulator of stemness, EMT and β-catenin in gastric CSC, thereby responsible for gastric cancer malignancy and drug resistance. All the data suggest NANOGP8 is a potential therapeutic target for cancer intervention. This is the first systematic study to show that NANOGP8, a human unique retrogene, is the key gene to determine gastric cancer malignancy.
